# Gleanings from Our Exchanges

**Published:** 1874-02-15

**Authors:** 


DR. BRUNELLI ON ELECTRO-THERAPEUTICS.—Dr. Brunelli (*La Independencia Medica*) has published the results of a three years' clinical treatment of nervous disease, at the Santo Spirito, in Rome. With respect to cerebral paralysis, of forty-two cases, eighteen were treated by Faradic electricity, eighteen with the galvanic current, and six by the mixed method.

Of the cerebral hemiplegias treated by Brunelli, those on the left side derived the less benefit from treatment—and especially was this the case where contractions were present frequently—whilst in the right side difficulty of speech was predominant, amounting, sometimes, to true aphasia.

In the second category, the author speaks of spinal palsies, of which, among these cases, he observed six monoparaplegias, and the rest double paraplegias; four of rheumatic origin (meningo myelitis); three produced by cerebro-spinal fever of short duration; three originating in circumscribed effusions of blood (all of which were of the character of monoparaplegia); two cases produced by gravileo-typhlitis; one by a wound; and the rest by inflammation, or parenchymatous myelitis, more or less acute. Many of these cases were cured by recourse to a mixed method.

The facial palsies, the rheumatic hysterical, or those caused by the traumatic lesion of the mixed nerves, which the author classifies among peripheric paralyses, have afforded numerous triumphs to electro-therapeutics. The gravest facial palsies cited by Brunelli were all cured, notwithstanding the age of some of these.

The author divides neuralgia into five divisions: there were five cases of sciatica, three brachial, two cervico - brachial, one of gastralgia, three lumbo-abdominal, one plantar, and one of sympathetic *tic*. The galvanic current, as usual, has given Dr. Brunelli satisfactory results; and in some of these cases the cure was very rapid, especially in rheumatic neuralgia.

With respect to cases of sclerosis in disseminated spots, ataxia, chorea, scriveners' palsy, the author does not seem to register any cure of these cases, although some were much alleviated by treatment.

Anæsthesia accompanied with pain, and simple anæsthesia, offer some cases of cure in Dr. Brunelli's clinique; and one case of ambliopia of the right eye is mentioned, with anæsthesia of the corresponding half of the scalp, cured by the galvanic current.

Idiopathic or peripheral contractions, according to the author, form the seventh category, of which he had only three cases; and in the eighth category, he presents us with different cases of convulsions, among which are epileptics. Of thirteen epileptics treated by the galvanic current, he cured two; three were notably benefited, and the rest remained *in statu quo*. The author used, for galvanization of the brain, a Daniell's battery of from six to ten elements.

In the numerous patients affected with arthritis, whether simple or multiple, rheumatic or traumatic, the galvanic current produced those excellent results expected from it in such cases; so that Dr. Brunelli believes that many other inflammations, such as the rheumatic and traumatic inflammation of the joints, ought to be treated in this way. In cases where there is muscular atrophy of the affected limb, the author prefers to raise the nutritive powers by galvanization of the sympathetic. From the cases of progressive muscular atrophy treated by Dr. Brunelli, and the slight results obtained in this affection, he deduces that electricity is but slightly efficacious in combating this terrible disease. As little confidence has he in the treatment of pseudo-hypertrophic palsies.—*The Doctor*, Jan. 1st, 1874.

---

BILIOUSNESS.—Some day we may arrive at definite ideas respecting the conditions included under this term. At present, it is employed to mean almost any derangement of the chylopoetic process. In consequence of some experiments lately made in Germany, by injecting cholesterine into the circulation of animals, Professor Austin Flint, jr., who had long previously worked at the subject, has re-stated his views before the New York Academy (*Med. Record.* Dec., 1873). He says the elements of secretion do not pre-exist in the blood; but those of excretion do, and they are separated, not manufactured, by glands. He finds that cholesterine is always present in the blood, which gains twenty-three per cent. of this substance by passing through the brain, and loses as much by passing through the liver. He concludes, therefore, that it is excrementitious—formed in the nervous system, and removed by the liver. If this organ becomes disorganized, it accumulates in the blood; and the term cholesteræmia is justifiable. Having been separated by the liver, the cholesterine in the bile passes into the intestines, and there changes into stercorine, of which some ten grains daily are discharged.

Dr. Barker alluded to the several sluggish conditions termed "biliousness," and which had also been called "cholæmia," though they have never been properly explained. But, if cholesterine be really the effete *debris* of nerve-tissue, we can certainly understand the torpor, headache, and some other symptoms that appear to arise in over brain-work. Dr. Barker, too, has found convulsive cases sometimes depend, not on uræmia, but, perhaps, on cholesteræmia; and he has been successful by diverting attention to the liver, rather than the kidneys. Dr. Barker's name is a sufficient guarantee for his clinical facts; and it appears to us that the question of acting upon the liver is still one to be entertained, or, at any rate, that imperfect function of that organ may give rise to disease.—*The Doctor*.

---

EMPYEMA — PARACENTESIS THORACIS — RECOVERY.— M. H. Alderson, M.D., of Bath, Ky., communicates this case: "Philo Howe, twenty-seven years of age, and of rather delicate constitution, was attacked, February 25th, with right pleuro-pneumonia. Convalescence was established in two weeks, and I did not see him again until the 1st of April, when I found him suffering from great distention of the right pleural cavity; the right side of the chest was œdematous, as well as the feet and legs. Hydragogues and diuretics were administered, without benefit; and as the oppression in breathing was so great, on the 30th of April, assisted by Dr. Shaw, of Bath, I performed paracentesis, giving exit to six pints of pus. Tonics and stimulants were directed. On the 5th, and also on the 16th of May, the tapping was repeated, the discharge each time being as great as at the first. The last time, before withdrawing the canula, I introduced a rubber catheter through it; then withdrew the canula, bent the external portion, and fastened it, by adhesive plaster, to the chest, and thus secured a continuous drainage for the fluid of the cavity. The discharge, varying in quantity from a few ounces to a pint daily, continued until June 20th, when, as it was scanty and entirely serous, I withdrew the catheter, and the aperture soon closed. Since then the man has been able to work as a farm-hand, and seems entirely well." — *American Practitioner.*

———

ELECTRICITY IN PARTURITION.— Dr. Ulisse Martemucci (*Lo Sperimentale*) says that already, in 1871, he had used electricity as an important assistant in cases of labor with uterine inertia, when ergot of rye had failed. The method consisted, in one case, in applying the induced current, placing one electrode, with a moist sponge, on the right side of the abdomen, at the level of the umbilicus, and the other also, with a moist sponge, on the left, running over the abdominal muscles, first with the one and then with the other. In fifteen minutes the fœtus was expelled, dead, without the use of forceps, which are always dangerous. In the *Gazzetta di Torino*, 1873, he reports two other similar cases, in both of which the children were born in the best condition of health. Also, in eight other cases, he succeeded without the use of ergot. He observes that the labor takes place much more rapidly thus than when ergot is used, and that, in these eight cases, he never lost one fœtus; whilst with ergot he lost one in four. Hence, he derives the following corollaries: 1. By using electricity, the obstetrician has in hand a method of causing the cessation of uterine contractions whenever he chooses; whilst, when ergot is used, the action is constantly kept up. 2. When ergot is used, it is necessary that the labor should be speedily finished, on account of the fœticidal properties of the drug, because the fœtus and placenta are so comprised as to make circulation difficult. 3. By the electric current, also, the obstetrician can leave off by turns and again recommence the uterine contraction, which he cannot do in cases where ergot is used.—*The Doctor.*

———

PEMPHIGUS.—Picot (*Jahresbericht Gesammten Medicin*, 1873, from *Gaz. des Hop.*) strongly recommends the treatment introduced by Hillairet, and which resembles that for burns, described in the last semi-annual report. It consists in applying to the affected skin, bandages soaked in a liniment of oil and lime-water. In the two cases reported by him, the bullous eruption extended over nearly the whole body, and was accompanied by severe itching. The fever was considerable. Both patients were bound up, from head to foot, in wadding, soaked in the preparation, which was daily changed. The general condition improved, the temperature sank without internal medication, and, later, the fever entirely disappeared. The excoriations, arising from the bursting of the bullæ, quickly dried, and healed in a short time. In one of the cases, no new bladders appeared after six weeks, while, in the other, perfect recovery only followed in two and a half months. In the latter case, a new eruption immediately followed a few days' interruption of the treatment. Hillairet has pursued this method for two years, in eight or ten cases, and always with similar results. In two cases of pemphigus foliaceus, it was less favorable.— *Boston Medical and Surgical Jour.*

———

GLANDULAR ENLARGEMENT IN DIPHTHERIA. — M. Bouchut regards this complication as adenitis, with diffused inflammation of the surrounding tissues. The pus is slow to collect into an abscess; and when this is found, there is always deeper mischief. Nevertheless, Bouchut recommends (*Bull. de Ther.*) early opening as the surest mode of cure. If necessary, he uses a drainage-tube. These remarks apply to enlarged cervical glands in scarlatina and croup, as well as diphtheria.—*The Doctor.*

DIAGNOSIS OF STONE.—Dr. Henry H. Head, physician to the Adelaide Hospital, reports a case, in the *Irish Hospital Gazette*, July 15, 1873, in which auscultation was employed as an aid to diagnosis of stone in the bladder. He says: "I sounded his bladder, and was pretty sure I detected a stone, but did not think the evidence absolutely conclusive, when it occurred to me to try auscultation, to see if it would assist my diagnosis. I accordingly applied one end of an India-rubber tube to the top of the catheter with which I was examining him, and the other to my ear, and at once heard, with great distinctness, the instrument strike the stone." He afterwards performed many experiments with substances of various sizes and degress of hardness, placed in a bladder distended with water, and found the sense of hearing to be more delicate than the sense of touch. "Even a small piece of chalk, not larger than a pea, could be most easily detected; the slightest touch of the catheter or sound being conveyed to the ear, when it could not be recognized by the hand." The stethoscope "consists of a small vulcanized India-rubber tube, about eighteen or twenty-four inches long, to one end of which an ivory ear-piece is attached, similar to that used for ear-trumpets; and into the other end is inserted a metallic plug, with a tapering end protruding, which should be pressed tightly into the canal of the catheter; or, if a solid sound is used, the end of the tube, without the plug, may be fastened to it."—*Boston Journal*, Dec. 26, 1873.

———

THE GALVANIC WIRE IN SURGERY.—Do British surgeons avail themselves sufficiently of this mode of bloodless section? This may be doubted; and when we seek for the reason we shall soon find that it lies principally in the trouble with which the use of the wire is connected. Now, however, that bloodless operations have become popular, it behooves all those who have become conversant both with galvanic apparatus and surgery to devise means of simplifying this operative measure. A few days ago Prof. Boeckell, of the faculty of Nancy, showed, at a meeting of the Surgical Society of Paris, an apparatus with which he can graduate the force of the current, and remove tumors without shedding a drop of blood. M. Trelat, at the same meeting, spoke in favor of the instrument, but found fault with its complicated appearance, and brought forward one made by M. Trouve, and modified by M. Onimus, which is simple, and acts very satisfactorily. There are a great number of operations in which the wire cautery should be used, so as to save the patient loss of blood. As Esmarch's method can only apply to the limbs, we ought to see that operations on the head or trunk be performed, when advisable, by the galvanic cautery, which promises to be almost as saving of human blood as Esmarch's proceeding.—*London Lancet.*

———

SIR H. THOMPSON ON LITHOTRITY.—In an address on the "Future of Operative Surgery for Stone in the Bladder," delivered before the Midland Medical Society, Sir Henry Thompson prophesies the universal success and applicability of lithotrity in the adult. He said, if the stone be not larger than an ordinary nut, requiring only two or three crushings, a perfect result may be insured; and that he had operated upon sixty-three such cases, of a mean age of over sixty years, with no fatality.

Sir Henry's first deduction is:

"That the diagnosis of the presence of stone in the bladder, and of its size, is of the highest importance."

The second:

"That the operation of lithotomy must. in future, be rejected for all stones which are of moderate size."

The moral of the address being, as we understand it, that as our knowledge increases, all stones may be discovered while small, and, therefore, all may be certainly cured by lithotrity.

For the discovery of small stones, the lecturer advised that the patient should make water a few minutes before sounding; and says that, in the whole course of his experience, he has not met with more than two or three cases in which the obvious early signs of calculus were absent.—*The Doctor.*

———

ASPIRATING PUNCTURE IN STRANGULATED INGUINAL HERNIA.—Case recorded by Dr. Albanese in *Gazetta Clinica di Palermo.*—Patient, thirty-seven, and suffering for three years from reducible inguinal hernia, suddenly presented signs of strangulation. Taxis was performed uselessly, and the worse signs came on: imperceptible pulse, fæcal vomiting, etc. The tumor was the size of a lemon, transparent, and sonorous on percussion. Taxis, after local and general anæsthesia, was again vainly tried. The mesial and external part of the tumor was then punctured. About four drachms of an alkaline liquid, without any smell, came away. Some gas escaped; reduction was not possible. The instrument was then introduced about one inch higher. Five drachms of fluid were then aspirated, and more gas escaped. Taxis became possible, and the patient soon recovered.—*London Lancet.*

REMEDY FOR CHRONIC HOARSENESS.—An eminent physician of Philadelphia contributes the following: In chronic hoarseness, arising from thickening of the vocal chords and adjacent membrane, the ammoniated tincture of guaiacum is often a very efficacious remedy. It may be appropriately mixed with equal parts of the syrup of senega, and a teaspoonful of the mixture given two or three times a day.—*Amer. Prac.*

———

REST IN LOCOMOTOR-ATAXY.—In the July number of the *American Journal of Medical Sciences*, Dr. Weir Mitchell insists on the great benefit of rest in the above disease. In cases of locomotor-ataxy in which the occurrence of various accidents, such as fracture of a leg, had compelled the patients to take absolute rest in bed during some time, the symptoms. and especially pain, were considerably amended, and in some instances the course of the disease was impeded or slackened. One case was experimentally conducted. A sufferer from an intense attack of the disease was subjected to absolute rest, without any other kind of treatment, and considerable amendment of all the symptoms was the result.—*London Lancet.*

